# Different Microfluidic Environments for In Vitro Testing of Lipid Nanoparticles against Osteosarcoma

**DOI:** 10.3390/bioengineering8060077

**Published:** 2021-06-04

**Authors:** Oihane Mitxelena-Iribarren, Sara Lizarbe-Sancha, Jay Campisi, Sergio Arana, Maite Mujika

**Affiliations:** 1 CEIT-Basque Research and Technology Alliance (BRTA), Manuel Lardizábal 15, 20018 Donostia-San Sebastián, Spain; saralizarbe93@gmail.com (S.L.-S.); jcampisi@regis.edu (J.C.); sarana@ceit.es (S.A.); mmujika@ceit.es (M.M.); 2 School of Engineering at San Sebastián, Universidad de Navarra, Manuel Lardizábal 13, 20018 Donostia-San Sebastián, Spain; 3 Department of Biology, Regis University, Denver, CO 80221, USA

**Keywords:** lipid nanoparticle, methotrexate, encapsulation matrix, tumor-on-a-chip, microfluidic platform, drug delivery, pediatric cancer therapy

## Abstract

The use of lipid nanoparticles as biodegradable shells for controlled drug delivery shows promise as a more effective and targeted tumor treatment than traditional treatment methods. Although the combination of target therapy with nanotechnology created new hope for cancer treatment, methodological issues during in vitro validation of nanovehicles slowed their application. In the current work, the effect of methotrexate (MTX) encapsulated in different matrices was evaluated in a dynamic microfluidic platform. Effects on the viability of osteosarcoma cells in the presence of recirculation of cell media, free MTX and two types of blank and drug-containing nanoparticles were successfully assessed in different tumor-mimicking microenvironments. Encapsulated MTX was more effective than the equal dose free drug treatment, as cell death significantly increased under the recirculation of both types of drug-loaded nanoparticles in all concentrations. In fact, MTX-nanoparticles reduced cell population 50 times more than the free drug when 150-µM drug dose was recirculated. Moreover, when compared to the equivalent free drug dose recirculation, cell number was reduced 60 and 100 points more under recirculation of each nanoparticle with a 15-µM drug concentration. Thus, the results obtained with the microfluidic model present MTX-lipid nanoparticles as a promising and more effective therapy for pediatric osteosarcoma treatment than current treatment options.

## 1. Introduction

Osteosarcoma, an uncontrolled growth of malignant cells produced in immature bone tissue, is one of the most common types of cancer between 1 and 14 year-old children [1]. This type of cancer is usually detected late in children, as its symptoms are often misdiagnosed as growing pains. High dose methotrexate (HD-MTX) was traditionally a key drug in the therapy of this type of cancer. However, these chemotherapeutics have several adverse side effects due to their indiscriminate distribution throughout the whole body [2].

In light of these side effects, novel therapies were developed in order to offer an efficient and targeted treatment avoiding unnecessary damage to healthy tissue. The use of different nanovehicles to transport drugs and deliver them in a controlled way minimizes drug degradation or loss. This approach prevents the side effects that the dispersed drug can cause and increases drug availability to the required sites [3,4]. Among these nanovehicles, nanoparticle-based drug delivery systems have been shown to increase drug accumulation at tumor sites, as well as improve permeability and retention of the drug [5]. Moreover, nanoparticles have been demonstrated to improve pharmacokinetic profiles and reduce side effects [6,7]. Therefore, the use of nanoparticles appears to be a promising strategy in cancer treatment [8,9].

In this context, lipid nanoparticles can act as therapeutic vehicles for antineoplastic drugs and are a highly potential alternative for the oral administration of anticancer drugs [10,11]. Moreover, lipid nanoparticles represent a more convenient and less invasive treatment for children, as they avoid the intravenous route [12,13]. These lipid nanoparticles help the drug absorption in the intestine and are orally administered averting the parenteral via, therefore, increasing patient comfort [12,14]. Once inside the body, they enter into the systemic circulation and, due to either the enhanced permeability and retention (EPR) effect or to an active targeting by surface modifications [15,16], lipid nanoparticles get straight to the specific site of action. This way, they release the drug in the affected area and increase the bioavailability inside the tumor [17]. Their delivery efficacy was demonstrated to be successful with different drugs, such as edelfosine [14,17,18], doxorubicin [19,20,21] and curcumin [22,23]. In these studies, it was shown that lipid nanoparticles had good biocompatibility, high encapsulation efficacy, excellent stability, sustained drug release, high tumor specificity and, therefore, less toxicity.

Several types of lipids, solid at both room temperature and human body temperature, were used in the development of solid lipid nanoparticle (SLN) [24,25]. The most popular ones are purified mono, di and triglycerides or fatty acids. The lipid phase is frequently composed from various chemical compounds and, therefore, the stability of the drugs may differ among the lipids used. Consequently, lipids are stabilized by mixing them with suitable surfactants [24,26,27], which guarantee the proper formation of SLNs, ensuring their stability and controlled drug release. It was demonstrated that a higher concentration of surfactants in the SLN preparation resulted in a decrease in particle size, which is desirable for intravenous administration [28]. Some of the surfactants used in SLN fabrication are non-ionic surfactants, anionic surfactants, alcohols or bile salts. Furthermore, some lipids, such as phospholipids, are used as surfactants too. Lecithin (LEC), the most remarkable compound of this last group, is a group of amphiphilic fatty substances occurring in animal and plant tissues, such as egg or soy. It was widely used in the fabrication of SLNs, demonstrating to be a great emulsifier of the mixture [29,30,31,32,33,34,35,36,37,38,39,40,41,42,43,44,45,46,47]. The other two most common surfactants are: Polysorbate 80 (also called Tween 80), which is a viscous water-soluble yellow liquid which hydrophilic groups contain a polyoxyethylene group; and Polyvinyl alcohol (PVA), a water-soluble synthetic polymer with excellent emulsifying properties and resistance to oil, grease and other solvents. Both were widely used in the formation of SLN [33,34,37,47,48,49,50,51,52,53,54,55,56,57,58].

In vitro analyses of the described vehicles were widely performed using traditional techniques. However, these traditional in vitro techniques offer a limited potential due to fluid stagnation. In fact, according to previous studies [18], static assays performed with blank lipid nanoparticles and methotrexate-lipid nanoparticles did not demonstrate significant difference in their cytotoxic effect, as their IC_50_ was found to be 11 µM and 10.23 µM, respectively. In fact, nanoparticles above certain concentration ranges deposited onto the bottom of the wells, killing the cells by asphyxiation, preventing the determination of the real effect of the treatment. As shown in the literature, the use of microfluidics in the biomedical field was of great utility in the development of alternative technologies for dynamic biomedical applications, mimicking several organ microenvironments [17,59,60,61,62,63,64,65,66]. In fact, microfluidic devices offer several advantages in mimicking biological systems, such as sizes similar to the biological length scales. Moreover, microfluidic devices offer highly-controlled fluidics, channel geometry and microenvironment control and the real-time monitoring of the process inside the device [67]. Moreover, microfluidics exhibit other advantages in biomedical applications, such as improved sensitivity over other techniques, time efficiency, reduced reagent and sample volumes and costs. They also allow multi-parameter studies on cancer cells, providing rapid analysis of small amounts of patient derived cells [68]. Microfluidic platforms fabricated with polymeric substrates offer new alternatives for the in vitro characterization of the effect of nanoparticles [69], which is also demonstrated in the current series of studies.

Considering all the above-mentioned facts, the effect on osteosarcoma treatment of methotrexate (MTX), encapsulated in different matrices, was assessed. The cytotoxic activity that free MTX and two different MTX-loaded lipid nanoparticles (named LEC-PVA and LEC-Tween) had against human osteosarcoma cells was analyzed in different concentrations and under dynamic conditions for the first time. Different microfluidic designs were used to perform the cytotoxicity analyses, mimicking the tumor microenvironment. The different microfluidic characteristics, such as the chamber height, were experimentally tested prior to the cytotoxic analyses. The different platforms utilized in this study probed if MTX-lipid nanoparticles are more effective at killing osteosarcoma cells than free drug treatment.

## 2. Materials and Methods

### 2.1. Design and Fabrication of the Microfluidic Platform

In these studies, a previously optimized CAD software design (AutoCAD^®^ 2015, Autodesk Inc., San Rafael, CA, USA) of the microfluidic platform was used. These microfluidic devices include: (1) two inlets to independently perfuse the cells and reagents/treatments into the platform; (2) two outlets to remove the waste; (3) a detection zone or chamber to analyze the cells in each study and (4) channels connecting the inlets/outlets with the chamber. The channel width was gradually increased from the inlets to the chamber in order to reduce the cell and nanoparticle velocity inside the latter. Design details are further described in our previous work [66].

In order to analyze different microenvironments, the platforms were fabricated in three different heights: 100 µm, 50 µm and 25 µm. Based on previous results from our group [66,70] and others [71,72,73], the microfluidic system fabricated with a height of 100 µm was used as a reference. Heights were tested to analyze whether the approximation of the nanoparticles to the cells at the bottom of the devices enhanced platform performance. The three platforms were fabricated in Polydimethylsiloxane (PDMS, Sylgard 184, Dow Corning) and glass. PDMS replica-molding techniques were performed in order to obtain the microfluidic platform: molds fabricated by conventional UV photolithography on 4″ silicon wafers with SU-8 100 photoresist were used, as previously described [74]. After the PDMS was demolded, an oxygen plasma treatment was used to make both the glass slide and the PDMS surface hydrophilic and provide a strong and irreversible bonding between them.

### 2.2. Reagents for Cell Culture and MTX-Lipid Nanoparticles

The U-2 OS (ECACC 92022711, ATCC HTB-96) osteosarcoma cell line was used in this study. Cells were cultured using RPMI 1640 with Ultraglutamine 1 (RPMI 1640 with U1, Life Technologies, Cramlington, UK) supplemented with 10% (*v*/*v*) fetal bovine serum (FBS, Life Technologies, Cramlington, UK) and 1% of PenStrep (Gibco^®^, Thermo Fisher Scientific, Waltham, MA, USA) at 37 °C in a humidified 5% CO_2_ atmosphere. For subculturing, cells were washed with PBS, trypsinized with 0.5% trypsin-EDTA 1X (Gibco^®^, Thermo Fisher Scientific, Waltham, MA, USA) and, once mixed with supplemented medium, centrifuged at 1500 rpm for 5 min. After this procedure, cells were used for subculturing (performed three times a week using conventional protocols) or seeded in the microfluidic devices for the experimental procedures.

Free MTX (kindly provided by Dr. A. Aldaz, from the Department of Pharmacy of Clínica Universidad de Navarra, Spain) was used as reference treatment to assess the effectiveness of the encapsulated MTX in three different concentrations: 15 µM, 150 µM and 1.5 mM (Table 1).

Two different types of lipid nanoparticles (kindly provided by Dr. M. J. Blanco, from the Faculty of Pharmacy of the University of Navarra) were subjected to study, named LEC-PVA and LEC-Tween. Each type was used in both blank and MTX-loaded conditions. The fabrication was previously described by González-Fernández et al. [18]. Briefly, both blank and MTX-lipid nanoparticles were prepared according to the hot homogenization method followed by high shear homogenization and ultrasonication, previously patented by the same group with minor modifications [17]. Before every in vitro experimental procedure, the dried lipid nanoparticles were suspended in cell media at the required MTX concentration (15 µM, 150 µM or 1.5 mM) and warmed in an ultrasonic bath at 37 °C for 4 min. The average size of the nanoparticles before lyophilization was 266 ± 18 nm for the LEC-PVA and 122 ± 6 nm for the LEC-Tween and their PDI values were 0.185 and 0.232, respectively. LEC-PVA nanoparticles had a drug-load of 25.93 ± 1.4 µg MTX/mg nP, whereas the drug-load of LEC-Tween nanoparticles was 19.84 ± 0.6 µg MTX/mg nP. Therefore, the number of nanoparticles used in each experiment changed according to the drug concentration that was tested, following the quantities shown in Table 1.

### 2.3. Experimental Set-Ups and Procedures

Before conducting the cytotoxicity tests to verify nanoparticle efficacy, cell velocity analyses were performed inside the different platforms.

First, the liquid and particle velocity profiles inside the microfluidic platforms were experimentally determined. Cells were inserted and circulated through the device, to determine the effect of the microfluidic platform height. The experiments were recorded with a Nikon Eclipse TS100 microscope and velocities of the cells were quantified with the “CellTracker” program extension of Matlab (The Mathworks, Inc., Natick, MA, USA). The maximum and non-zero minimum velocity values were experimentally calculated for each height. 

**Figure 1:**
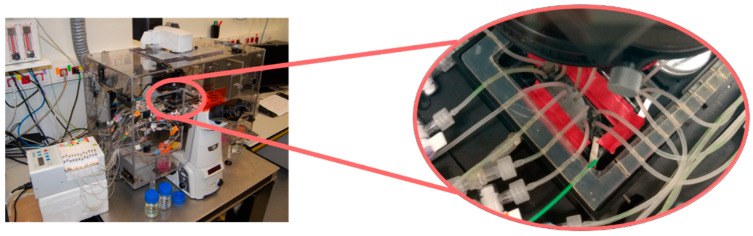
Experimental set-up including a transmission Nikon Eclipse Ti microscope, with a high-resolution monochrome Hamamatsu camera and a specific stage, which allowed the control of temperature and environmental conditions to perform cytotoxicity assays.

Next, cytotoxicity tests were conducted in a set-up including a transmission Nikon Eclipse Ti microscope, with a high-resolution monochrome Hamamatsu camera and a specific stage, which allowed the control of temperature and environmental conditions (Figure 1). 

For all experimental procedures, cell media was circulated inside the chambers to interact with the PDMS surface, filling the platforms and providing an environment more prone to cell adhesion. Cells were then inserted in the microfluidic chambers in a density of 7 × 10^4^ cells/µL and were allowed to adhere onto the bottom of the devices. In order to evaluate the cytotoxic effect of the different treatments subjected to study, the following assays were performed with different height platforms over the cell monolayer:

- No treatment (1), keeping cells under recirculating media in order to determine their normal proliferation.
- Regular treatment (2), where cells were subjected to recirculating free MTX to determine the cytotoxic effect of the circulation of different drug concentrations.
- Nanoparticle controls (3), namely cells were kept under the recirculation of nanoparticles with no MTX inside (blank nanoparticles). In this case, both LEC-PVA and LEC-Tween type blank nanoparticles were used.
- Nanoparticle treatments (4), where cells were subjected to the recirculation of either LEC-PVA or LEC-Tween encapsulated MTX in different concentrations.

For the assays entailing the use of MTX (2) and (4), three drug concentrations were considered: 15 µM, 150 µM and 1.5 mM. Assays (1) and (3) were carried out as controls. In the case of “Nanoparticle controls”, the nanoparticle concentration was given by that used in the corresponding “Nanoparticle treatment”. The calculation of the nanoparticle mass required in the latter was based on the amount of drug trapped per nanoparticle mg. As performed in previous studies [66], all cell assays consisted of four steps: (i) cell insertion, (ii) cell adhesion under static conditions for 24 h, (iii) cell proliferation under corresponding treatment, and (iv) image acquisition after 12, 24, 36, 48 and 72 h, to evaluate the cytotoxic effect without stopping the treatment application. Recirculation of the treatments was performed with the use of an IPC peristaltic pump (Ismatec) at 2.15 µL/min. The same procedure as described previously [66] was used in order to determine the effectiveness of the different treatments. Briefly, the number of cells remaining in the platform was quantified for every condition in every time point considered. These adherent cells remained attached and with a specific flat shape if they were still alive at the end of the assay, which was verified using Live/Dead fluorescent dies. On the other hand, dying cells developed vacuoles and plasma membrane blebbing, becoming round before they finally detached from the bottom of the platform and went with the flow. Only dead cells (which burst when they detached) were removed from the chamber with the liquid flow. Therefore, the number of living cells changed with the disappearance of the dead cells.

### 2.4. Statistical Analysis

The non-zero minimum and maximum velocity values obtained were statistically analyzed using Matlab (The Mathworks, Inc.). Data analysis was carried out by performing independent student’s *t*-test when comparing the different heights. Following the same procedure, statistical analysis was also performed to analyze the cytotoxicity effect using Matlab (The Mathworks, Inc.). When comparing only two groups, independent student’s *t*-test (or Mann–Whitney-U tests) was performed. One-way analysis of variance (ANOVA) followed by post-hoc Bonferroni’s test or its non-parametric equivalent test (Kruskall–Wallis test) was performed when comparing more than two groups. Statistical difference was determined as not significant (*p*-value > 0.05), significant (0.01 < *p*-value < 0.05), very significant (0.001 < *p*-value).

## 3. Results and Discussion

### 3.1. Cell Velocity during Insertion

Experiments performed with the three heights allowed for the estimation of the velocity of the liquid, cells and nanoparticles while entering the chambers. In previous studies, it was determined important to have the lowest nanoparticle velocity possible in the chamber to maximize the contact time between the circulating nanoparticles and the adhered cells, ensuring that there was no nanoparticle sedimentation throughout the treatment [66]. Thus, the obtained non-zero minimum and maximum velocity values were used to compare the influence of the height.

In respect to the 25-µm high platforms, experiments demonstrated that the cross-section area was not enough to allow the circulation of all the cells, because the number of cells that got inside the chamber was much lower than the ones entering the other chambers. This is because cells have a radius of around 20 µm and, therefore, not as many cells as in the higher platforms can be harbored. Moreover, the cells that entered the platform reached extremely high velocity values, which were too high to perform latter image analysis. Therefore, these platforms were discarded for further cytotoxicity studies, as they could not mimic an appropriate tumor environment.

Comparing the remaining two microfluidic devices of different heights, the highest maximum velocity value was obtained with the 50-µm channels at a rate of 2.22 ± 0.35 mm/s. This turned out to be nearly double the maximum velocity value obtained inside the 100-µm channels (1.12 ± 0.17 mm/s). The same proportion was observed with the non-zero minimum values, which were 0.52 ± 0.95 mm/s and 0.29 ± 0.13 mm/s, respectively. The increase in the velocity values can be explained by Bernoulli’s principle, which states that an increase in the speed of a fluid occurs simultaneously with a decrease in pressure or a decrease in the fluid’s potential (proportional to the height of the channel). This can be clearly represented with Bernoulli’s equation:

(1) $$\frac{v^{2}}{2}+gz+\frac{p}{\rho }=constant,$$

where, *v* is the fluid flow speed, *g* is the gravity, *z* represents the elevation of the point above a reference plane, *p* the pressure at the chosen point and *ρ* the density of the fluid. The highest velocity values were always found in the connection between the end of the channel and the entrance to the chamber, while the minimum ones were located on the sides of the chamber, closer to the walls rather than to the center.

When comparing these experimental values among them, an independent student *t*-test was performed. In this case, a very significant difference (*p*-values < 0.003) was observed between the different heights, confirming the effect of the chamber height in particle velocity and therefore in cell response to the treatment.

### 3.2. Treatment Comparison: In Vitro Cytotoxicity

**Figure 2:**
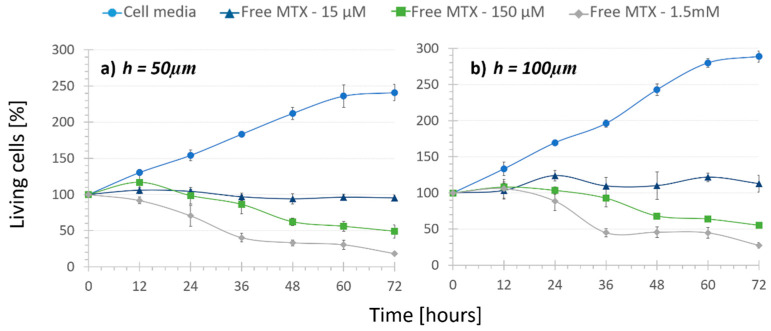
Cell viability under the recirculation of cell media and different concentrations of free MTX inside channels that were (**a**) 50 µm high and (**b**) 100 µm high (n = 4). Cell population increases with cell media recirculation, but decreases with an increasing amount of free drug recirculation.

In order to assess both microfluidic platforms to quantify the cytotoxic effect of drug-loaded nanoparticles, cells were first subjected to regular cell media circulation (i.e., “No treatment”) assays. These studies intended to prove that cells could withstand damage caused by the friction of continuous fluid flow. As depicted in Figure 2 with circular markers, cells proliferated as expected and withstood damage caused by the continuous flow rate during the 72-h period. Thus, the rubbing induced by the low circulation rate of 2.15 µL/min had nearly no damaging effect in the cells in any of the channel height platforms. However, it is noteworthy to remark that in the case of 50 µm devices, cells proliferated somewhat slower (approximately obtaining a 40-point lower cell population). This indicates a higher mechanical stress exerted on the cells due to a lower chamber height, which can also be deduced from Bernoulli’s principle, previously mentioned. However, this cell growth difference provoked by the height change was not statistically significant (*p*-value = 0.055).

Next, “Regular treatment” assays were performed to verify that inside the microdevice the increasing amount of MTX had an increasing cytotoxic effect. The three mentioned concentrations of the free drug were recirculated in both height channel platforms. Although it was statistically not significant (*p*-values > 0.08), it can be inferred from the free MTX curves (Figure 2, triangle, square and diamond markers) that under the same drug concentration, cell population was slightly lower when the channel height was smaller. For example, under 150-µM free MTX recirculation cell population was reduced to 48% in 50-µm high platforms and only to 55% in 100-µm high platforms. These values support the results observed in “No Treatment” assays. On the other hand, a cytotoxic effect of the recirculation of free MTX was observed. Cells under 15 μM free MTX recirculation in both height platforms stopped proliferating and the population remained around 100%. This demonstrated a cytostatic but not cytotoxic effect of the free drug with this concentration. Moreover, the increasing amount of MTX concentration from 15 µM to 150 µM and 1.5 mM provoked that after 72 h, cell population was reduced from 112% to 55% and 27% in the 100-µm high channels and from 95% to 48% and 28% in the 50-µm high channels, respectively. As expected, the use of different concentrations demonstrated a statistically very significant cytotoxic effect (*p*-values < 0.002), independent from the chamber height. Therefore, the dose-dependent chemical effect of the drug was proven inside the microfluidic platform, making it a good tumor-mimicking environment to test new treatments.

In the following step, lipid nanoparticles were recirculated over the cells. As described before, cells were subjected to blank lipid nanoparticles to assess the effect of adding a vehicle alone as a treatment. It must be mentioned that for both lipid nanoparticles, the amount of vehicle needed to obtain the 1.5 mM concentration of the drug was too high, distorting the image such that cells could not be distinguished to make the quantification. Moreover, the friction provoked by this mass detached the cells from the surface and dragged them with the flow, leaving nothing to analyze. Therefore, this concentration was discarded for the study.

**Figure 3:**
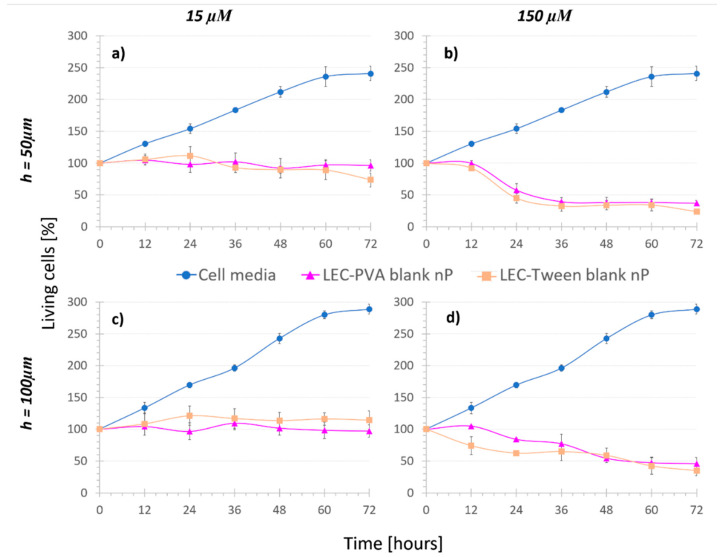
Cell viability under the recirculation of cell media and “Nanoparticle control” assays, where (**a**,**b**) are for 50-µm high platforms and (**c**,**d**) for 100-µm high platforms (n = 4). The recirculation of blank lipid nanoparticles reduced the cell population when increasing the concentration.

In the “Nanoparticle control” assays (Figure 3), cell population did not grow as much as under cell media recirculation. Considering both LEC-PVA and LEC-Tween blank nanoparticles, no statistical difference was observed between their effects comparing both channel heights (*p*-value > 0.4). Thus, again, channel height reduction had no remarkable damaging effect in cell population when recirculating both blank lipid nanoparticles.

Concerning concentration, under recirculation of both LEC-PVA and LEC-Tween blank nanoparticles, cell population was reduced over time for increasing nanoparticle concentrations, due to the mechanical stress caused by the higher number of vehicles. When recirculating the equivalent nanoparticle amount of 15 µM of both LEC-PVA and LEC-Tween blank nanoparticles, after 72 h, cell populations remained around 96% and 74% in the 50-µm high platforms and up to 97% and 115% in the 100-µm high platforms, respectively. However, when using the equivalent of a 150-µM dose these percentages were remarkably reduced for both height platforms: to 45% (LEC-PVA) and 35% (LEC-Tween). This indicates that cells died due to the higher stress caused by the recirculation of an increasing number of nanoparticles. It must be noted that no statistically significant difference (*p*-value > 0.05) was observed between LEC-PVA and LEC-Tween blank nanoparticles since both kept the cell population around the same percentages.

**Figure 4:**
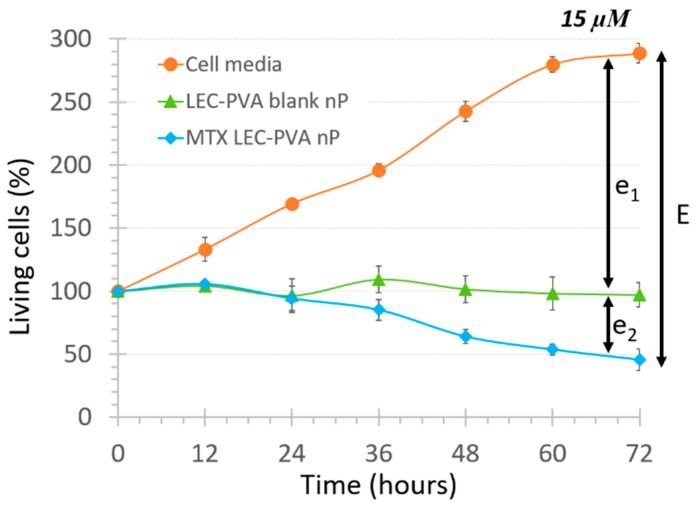
Total cytotoxic effect of LEC-PVA nanoparticles (nP) at 15 µM concentration inside 100-µm high platforms, showing the components e_1_ and e_2_ of term E defined in this study.

The “Nanoparticle control” assay demonstrated that there was a physical effect coming from just the recirculation of the nanoparticles above the cells. In order to distinguish the effect that the encapsulation of the drug itself had, a new variable was defined: the total cytotoxic effect (named E) of the MTX-loaded nanoparticles. This term was represented as follows:

E = e_1_ + e_2_, (2)

where e_1_ represents the physical effect provoked just by the recirculation of the nanoparticles and e_2_ represents the biochemical effect provoked by the internalization of the encapsulated drug itself. As an example of the effect observed with the blank lipid nanoparticles, the cell population decrease under the LEC-PVA nanoparticles under a 15-µM drug dose is shown in Figure 4.

**Figure 5:**
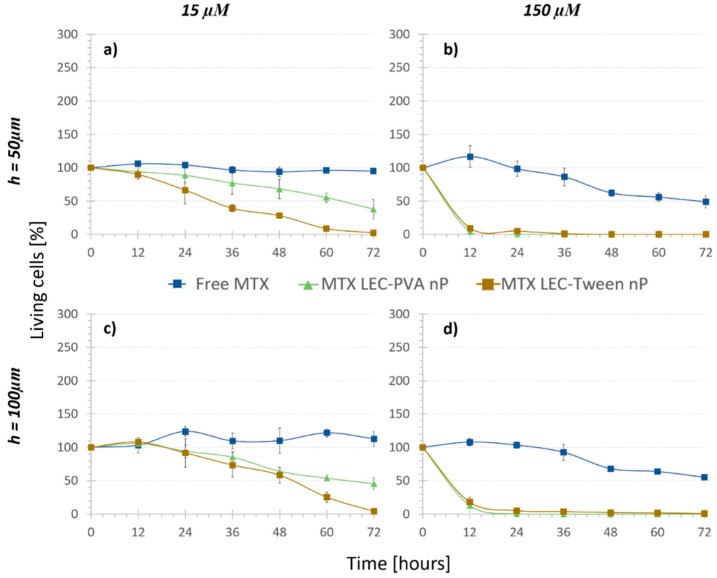
Cell viability difference between the encapsulated and free MTX at different concentrations inside (**a**,**b**) 50 µm high platforms and (**c**,**d**) 100 µm high platforms (n = 4). Both nanoparticles reduce the cell population faster than the free drug in both concentrations.

Finally, in order to assess and quantify the biochemical effect (e_2_) the “Nanoparticle treatments” were carried out. As shown by the dark triangle and square markers in Figure 5, cell growth was observed to be significantly reduced under the recirculation of both types of drug-loaded nanoparticles compared to that with the free drug. Indeed, it was demonstrated that the efficiency of encapsulated methotrexate was much higher than that of the free methotrexate: the higher effect of the loaded lipid nanoparticles against osteosarcoma cells was statistically significant already after 6 h under 150 μM encapsulated MTX concentration (*p*-values < 3 × 10^−7^) and after 24 h under 15 μM encapsulated MTX concentration (*p*-values < 1.55 × 10^−5^).

As mentioned before, in the “No treatment” and “Nanoparticle control” assays, the chamber height reduction from 100 μm to 50 μm caused no significant difference in the living cell population after 72 h. In the case of MTX-loaded nanoparticles, the damaging effect observed was also not noteworthy when comparing both channel heights (*p*-values > 0.35). Therefore, MTX-loaded nanoparticles reduced the cell population to similar numbers for the two heights. Even though the height change did not provoke a statistical difference in the results, when the cytotoxicity of the encapsulated and free MTX was compared, the statistical difference was usually observed earlier in the smaller chambers. For example, in the case of LEC-PVA nanoparticles at a 15 µM concentration, the cytotoxic effect was extremely significant (compared to that of the free MTX) from 48 h on inside the 100-µm high channels (*p*-value < 0.0001), while it was already significant after 36 h of treatment inside the 50-µm high channels (*p*-value < 0.03). Therefore, a cytotoxic effect was observed earlier in the smaller channels for the lowest concentration. However, this effect was not observed when comparing the cytotoxicity of both types of encapsulated MTX to the free one under the recirculation of a 150 µM concentration, as for both heights the cytotoxicity difference was extremely significant (*p*-value < 1 × 10^−6^) from the first time point on.

Following what was observed in the “Nanoparticle control” and “Regular treatment”, an increasing number of nanoparticles or drug concentration was translated into an increased reduction of cell population. This effect was observed with both types of MTX-loaded nanoparticles after 72 h of their recirculation. In the case of MTX-LEC-PVA nanoparticles, cell population was reduced from 45.7% to 0.9% when the drug concentration was increased from 15 µM to 150 µM in the 100-µm high platforms. Likewise, under the recirculation of MTX-LEC-Tween nanoparticles cell population was reduced from 4.2% to 0.5% when the drug concentration was increased from 15 µM to 150 µM in the 100-µm high platforms. In order to verify if this reduction was due to the friction of a higher number of nanoparticles or due to a higher amount of drug in the solution, the components of the above mentioned “E” effect term were analyzed for each nanoparticle. Both LEC-PVA and LEC-Tween nanoparticles demonstrated a higher physical effect than a biochemical effect in all the possible combinations of concentrations and platform heights. Moreover, when increasing the drug concentration from 15 µM to 150 µM, e_1_ increased as well. For example, under the recirculation of 15 µM treatments, LEC-Tween nanoparticles showed a physical effect of 70% of the total cytotoxic effect in 50-µm high platforms and 61% in 100-µm high platforms. These percentages increased to 90% and 88%, respectively, when the 150-µM treatments were recirculated (Table 2). 

Furthermore, focusing on the differences of cytotoxic effect of both types of MTX-loaded nanoparticles, both kept a similar viability trend under a 150-µM MTX concentration. The distribution of both physical and biochemical effects was not statistically significantly different: LEC-PVA nanoparticles demonstrated an e_1_ of 84% in both height platforms and LEC-Tween nanoparticles an e_1_ of 90% (50 µm) and 88% (100 µm) when the 150-µM treatments were recirculated. This observation reflects the similar physical effect that both nanoparticles had when the concentrations were high. Thus, this concentration was too high to ensure the evaluation of the effect that the nanoparticles could really have in the body. Moreover, it must be mentioned that once more, this comparison showed no significant difference between the results obtained from the 50-µm and 100-µm high platforms.

**Figure 6:**
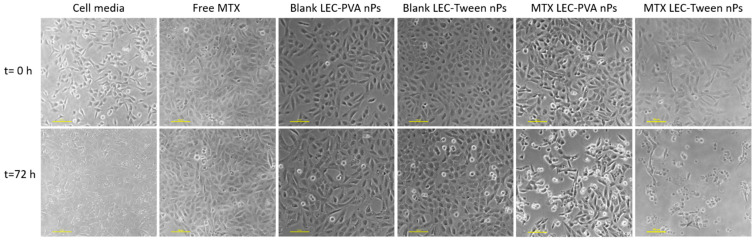
Cell viability difference between the beginning of the experiments and after 72-h of treatment recirculation, being the drug concentration of 15 µM and the microfluidic platform used of a height of 100 µm. Cells under media recirculation grow reaching high confluence. Free-MTX and blank nanoparticles have a cytostatic effect over cell viability, whereas MTX-loaded nanoparticles have a cytotoxic effect, reducing cell population. Scale bar: 100 µm.

On the other hand, the experiments demonstrated a higher toxicity rate for the LEC-Tween nanoparticles than LEC-PVA ones under a 15-µM drug concentration in both channel heights. Specifically, the 72-h recirculation of MTX-LEC-Tween nanoparticles reduced the cell population to 4.2% and 2.7% inside the 100-µm and 50-µm high platforms, respectively. Whereas, in the case of MTX-LEC-PVA nanoparticles, cell population was reduced to 45.7% (100 µm) and 38% (50 µm) in the same period. This demonstrated an extremely significant difference (*p*-values < 0.001) between the cytotoxic effects of both loaded nanoparticles. As an example of the treatment effect over cell cytotoxicity, Figure 6 shows the evolution of the cell population under cell media recirculation and the four different treatments. The pattern of cell viability observed in the different populations from the beginning to the end of the assay (after 72 h of treatment recirculation) demonstrates the differential responses to treatment over time.

According to the representation of this total cytotoxic effect (E), physical and biochemical effects of each nanoparticle type under the recirculation of 15 µM treatments reflected a significant difference in 100-µm high platforms. In the case of LEC-PVA nanoparticles, the physical effect went up to 79%, whereas for the LEC-Tween nanoparticles reached only the 61%. Therefore, LEC-Tween nanoparticles demonstrated a 39% biochemical effect, which was 18 points higher than the one obtained by the LEC-PVA particles. It is true that LEC-Tween nanoparticles had a lower drug-load than the LEC-PVA ones and, therefore, a higher amount was needed to have the same drug concentration. This could lead one to deduce that a higher friction could provoke a higher physical effect from them. However, their size was also smaller, which could make them more attractive for the cells and cause them to be up taken faster. On the other hand, it is well-known that Tween 80 and PVA have different physicochemical properties, such as molecular weight (131 g/mol for Tween 80 and 88% hydrolyzed Ww 124 Kda for PVA) and heavy atom count (42 for Tween 80 and 3 for PVA) [75,76]. The mechanisms with which the cells uptake the nanoparticles are the same in both cases (i.e., via endocytosis). Nevertheless, it was demonstrated that the material, size and stiffness of nanoparticles can affect the cell uptake: smaller and harder particles present enhanced uptake in both cancer and immune cells, for example [77,78]. The difference in material, size or stiffness between these two types of nanoparticles could provoke a different cell uptake. Therefore, these differences could explain the observation of a significant difference in cell population when comparing both loaded-nanoparticle treatments.

To sum up, it was proven that the channel height did not affect the cytotoxicity observed, although the smaller the height, the shorter the time for the treatment to show a significant toxicity difference. Furthermore, the nanoparticle quantity used in each assay was decisive: the higher the number of nanovehicles inserted in the microfluidic platform, the faster that a significant toxicity difference was observed. With these data, it was demonstrated that lipid nanoparticles had a higher nanoparticle efficacy compared to free drug efficacy, as under the same drug concentration, nanoparticles were shown to kill a higher number of cells in a shorter time. It was also demonstrated that with the use of lipid nanoparticles the amount of drug required to reduce cell population to half was 100 times lower than with the free drug. Therefore, nanoparticles would not only be a more specific, but also a less hazardous treatment for patients due to the drug dose reduction. Comparing both encapsulating matrixes, small differences were observed between both nanoparticles, when high concentrations where recirculated. On the other hand, when using low drug concentration values, although they had worse PDI values, LEC-Tween nanoparticles demonstrated to have a higher biochemical effect in osteosarcoma cells compared to the effect that LEC-PVA nanoparticles had.

## 4. Conclusions

The cytotoxic effect of different concentrations of encapsulated MTX in osteosarcoma cells was evaluated using a microfluidic device. Different microfluidic platform heights were tested in the study and velocities obtained inside each platform were experimentally analyzed.

In the current series of experiments, we demonstrate that dynamic microfluidic platforms are a promising alternative to current nanoparticle characterization assays for cancer treatment. No statistically significant difference was observed between the results obtained from the two channel heights. Nevertheless, a lower stress was obtained inside 100-µm high microfluidic platforms. Circulation of media demonstrated no damaging effect after 72 h in all platform heights, ensuring a good tumor-mimicking microenvironment. The increasing amount of free MTX recirculation had an increasing cytotoxic effect in the cells. Both LEC-PVA and LEC-Tween blank nanoparticles inhibited the cell population growth but kept it around 100% after 72 h in a 15-µM concentration. Cell population was reduced over time when the blank nanoparticle concentration was increased, due to the friction caused by the higher number of vehicles. Finally, cell growth was observed to be significantly reduced under the recirculation of both types of MTX-loaded nanoparticles compared to that of the free drug, demonstrating the higher effectiveness of the encapsulated MTX. When comparing both lipid nanoparticles, LEC-Tween nanoparticles under a 15-µM drug concentration demonstrated a higher toxicity rate than LEC-PVA ones. This could be explained with the analysis of the physical and biochemical effect that each nanoparticle had: LEC-Tween nanoparticles demonstrated a higher biochemical effect under the same drug concentration recirculation, which explains the higher toxicity.

Overall, the obtained results demonstrate that MTX-lipid nanoparticles are more effective at killing osteosarcoma cells than free drug treatment. That is to say that a 100 times lower drug dose would be required when treating with nanoparticles, which could potentially reduce several side effects to the patients, improving their quality of life. According to the encapsulating matrix, it was demonstrated that the use of different materials led to statistically different cytotoxic effects, provoked by their different physical and biochemical properties. Taken as a whole, the obtained results demonstrate that MTX-lipid nanoparticles are a promising and potentially more effective therapy for osteosarcoma treatment than current treatment options.

## Figures and Tables

**Table 1:** Free MTX and nanoparticle amount required per milliliter to obtain the desired drug concentration for each experiment.

MTX Concentration	Free MTX	MTX-LEC-PVA Nanoparticles	MTX-LEC-Tween Nanoparticles
15 µM	6.82 µg/mL	262.6 µg/mL	343.5 µg/mL
150 µM	68.2 µg/mL	2.626 mg/mL	3.435 mg/mL
1.5 mM	682 µg/mL	26.26 mg/mL	34.35 mg/mL

**Table 2:** Distribution of the physical (e_1_) and biochemical (e_2_) of MTX-loaded nanoparticles.

E	Nanoparticle Type	15 µM		150 µM	
		50 µm	100 µm	50 µm	100 µm
e_1_	LEC-PVA	72.4%	78.8%	84.7%	84.2%
	LEC-Tween	70.2%	61.3%	90.2%	88.1%
e_2_	LEC-PVA	27.6%	21.2%	15.3%	15.8%
	LEC-Tween	29.8%	38.7%	9.8%	11.9%

## Data Availability

The data presented in this study are available on request from the corresponding author.
